# Robotic Approach to Creation of Continent Catheterisable Channels—Technical Steps, Current Status, and Review of Outcomes

**DOI:** 10.3389/fped.2019.00001

**Published:** 2019-01-21

**Authors:** Ramnath Subramaniam

**Affiliations:** ^1^Consultant Paediatric Urologist, Leeds Teaching Hospitals NHS Trust, Leeds, United Kingdom; ^2^Department of Paediatric Urology, University of Leeds, Leeds, United Kingdom; ^3^Department of Paediatric Urology, University of Ghent, Ghent, Belgium

**Keywords:** catheterisable channel, robotic approach, pediatric urology, outcomes - health care, current status

## Abstract

**Purpose:** To report the current status of Robotic approach to creation of Catheterisable channel (CC) with the author's personal experience compared to published literature on technical steps, follow up, and outcomes.

**Methods:** CC data was extracted from the prospective database set up for all Robotic pediatric urology procedures performed by the author at his institution. A literature search was then performed to look at the evidence base.

**Results:** Eighteen consecutive cases (8M:7F) of Robotic approach to creation of CC was identified and included. All attempted cases were successfully completed without any conversion to open approach. Median age at surgery was 10.75 years (IQR 6.9–16.5); Median OT 197 min (IQR 131–295) with concomitant procedures in 4 cases. Appendix was used in 14 cases as CC conduit and distal ureter in 4 cases. Median Length of stay (LOS) was 2.75 days (IQR 2–6) and Median FU 27.3 m. Whilst FU duration is comparable to published series, average OT and LOS was much lower in this series. The LOS in this robotic series is much lower than the author's experience with open approach (2.75 vs. 5.8 days). No major complications postoperatively except for one exit site wound infection managed conservatively. None of the CC have been revised in this series and all channels are patent with 12 F or 14 F admissible catheter size. There were no cases of incontinence related to technique of creation of CC and no incidence of exit site stomal stenosis with use of ACE stopper until channel matures and Clean intermittent catheterisation (CIC) is established.

**Conclusion:** Robotic approach to CC is feasible, safe with excellent outcomes and minimum morbidity. Robotic complex bladder reconstructive surgery offers some advantages to children compared to open approach but is only currently performed in few tertiary centers with expertise.

## Introduction

Congenital and acquired affections to the bladder can lead to poor bladder emptying with attendant consequences of recurrent urinary tract infections, damage to renal upper tracts, and incontinence. Two vital landmark developments in the management of bladder emptying include Clean intermittent catheterization (CIC) per urethra introduced by Lapides in 1970s (1) and subsequent extension of this concept by Mitrofanoff to create catheterisable channel (CC) using appendix to facilitate bladder drainage when CIC per urethra is not feasible (2). Up until recently, open surgical approach was favored by most pediatric urologists with few reports of laparoscopic assisted attempt to create the channel. Robotics has recently provided an alternative with enhanced minimally invasive option in creating such channels with its attendant benefits (3, 4), not least to the surgeon due to ergonomics (5). This article looks at the author's personal experience with the Robotic approach, description of technical steps, and a review of published literature as regards potential benefits, current status and outcomes.

## Methods

The methodology in this paper is set in two parts. The first is the patient series from the author's personal experience and the second is the review of literature to ascertain outcomes when possible and define the current status of Robotic approach to CC comparing different studies along with the author's experience.

### Patient Database

A prospective database collating all information with regards to author's personal experience with Robotic approach to all procedures in pediatric urology was set up since 2013. Informed and written consent was obtained from the patients or their legal guardian in this study. Information related to creation of CC within this database was extracted and analyzed. A comparison to Length of stay (LOS) with similar group of cases with an open approach was made to calculate cost analysis together with patient level cost (PLICS) data from the author's institution. Formal ethical committee is not required for this type of audit.

### Literature Search

A search was done from Institutional library resources using terms “Robotic,” “Mitrofanoff” and “Catheterisable channel” and restricted to pediatric age group.

## Technical Steps in the Creation of the CC

The technical steps described below are the author's preferred approach recognizing there might be variations amongst other surgeons to the approach. At the outset, it is important to note that the author's preferred exit site for CC is right iliac fossa; some other surgeons might prefer the umbilicus. The robotic platform mentioned in this series is the Si Da Vinci system.

### Patient and Robot Positioning

Patient is placed supine on the operating table with extreme side docking of the robot from the left side. The right side therefore is available for exit site incision and placement of large step port to facilitate retrieval of the channel into the exit site. This assumes appendix or the right ureter as the chosen conduit for the CC. Side docking, often to the extreme is a useful maneuver in pediatric patients and has the advantages of leaving the patient supine on the table and the robot can be docked from either side (6). This is particularly important in small theaters where space can be a constraint given that existing theaters do not cater for the footprint of Robotic surgical equipment.

### Port Sites

**Figure 1:**
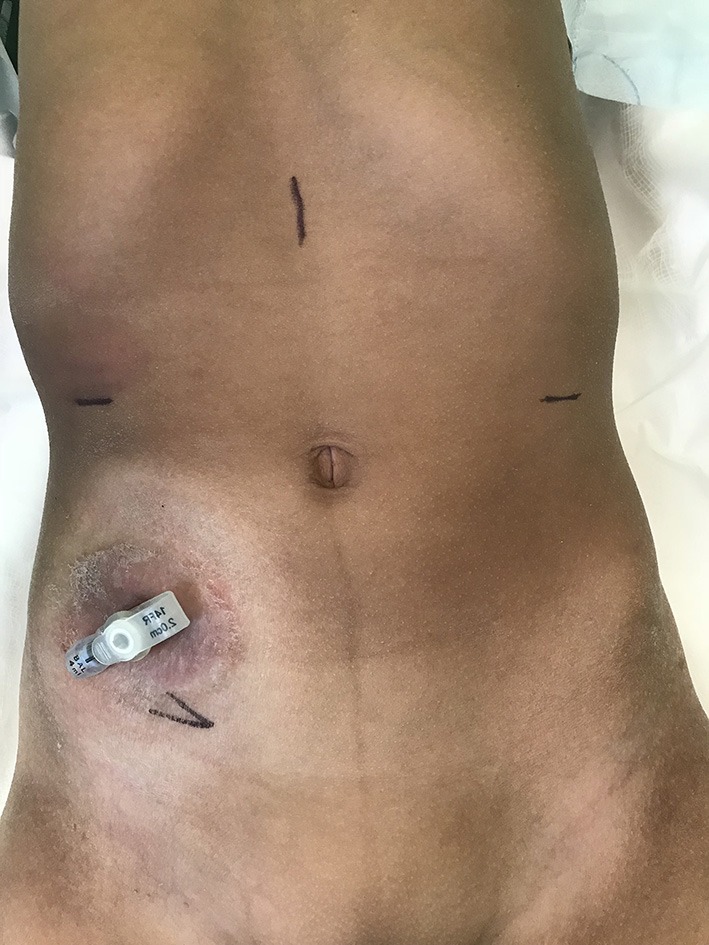
Preoperative picture of a patient with caecostomy button *in situ* for bowel washouts. Port sites are marked- 12 Camera port in epigastrium; two 8 mm working ports on the respective hypochondrium; Exit site at right iliac fossa.

The 12 mm camera port is placed in the midline of epigastric region to stay as far away as possible from target organs, namely the appendix and bladder. Two 8 mm working ports are placed in the right and left hypochondrium. The exit site is marked up with a “V”; this will eventually be used for the VQ plasty to facilitate skin lined stoma at exit site (Figure 1). During the procedure, a large (10–12 mm) step port is placed through the base of the exit site incision. This facilitates retrieval of the CC onto the exit site. Given the complex needs for these group of patients, particularly with bowel affection for e.g., Spina bifida cases, often they have a caecostomy in place for bowel management. In the author's institution, the caecostomy is fashioned with a button for washouts (if not effective per rectum). In the Figure 1, the child has a caecostomy button placed previously.

### Conduits for the Channel

It is generally agreed that appendix raised on its vascular pedicle is the best available conduit. The author has used the ureter in selected cases when a dilated tortuous ureter is available and there is no associated vesicoureteric reflux; when a nephroureterectomy has been performed for appropriate clinical indication or appendix is not available. The other option is to create a conduit with small bowel (Monti procedure); the author has no personal experience with this option by the Robotic approach. The outcomes from author's experience with appendix and ureter are detailed in the Results section.

### Procedure

**Figure 2:**
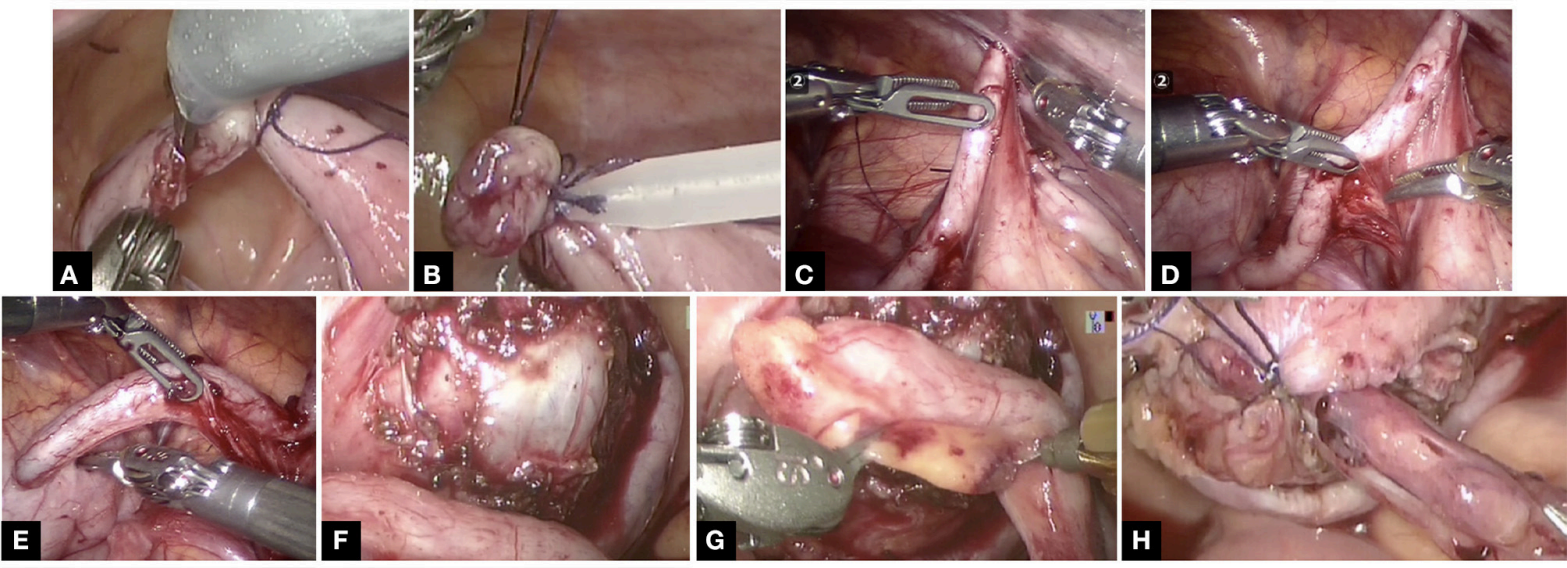
The technical steps in the creation of CC is shown in this series of pictures. Note not all pics are from the same case study but does represent the relevant individual steps in the procedure. **(A)** Appendix divided from caecum with ligature at caecal base. **(B)** Endoloop to secure the caecal stump further. **(C)** Appendix routed into exit site. **(D)** Ensuring vascular pedicle on its mesentery is not under tension and adequately mobilized. **(E)** Appropriately sized Foley catheter inserted into appendix. **(F)** Adequately sized detrusorotomy performed. **(G)** Typically, a 4:1 ratio of detrusorotomy to length of appendix ensures continence to channel. **(H)** Extravesical implantation of the appendix completed.

Once the ports are placed, the steps in creation of CC are illustrated in Figure 2. The appendix is mobilized on its vascular pedicle and divided from the caecum with a ligature and endoloop to secure the caecal stump. This is done through the step port placed by the assistant at the exit site. The appendix is then retrieved through the step port and an appropriately sized foley's catheter is placed into the lumen of the appendix. Typically, a 12 F catheter is placed, although in some cases, it might have to be a 10 F catheter, whilst some can accommodate 14 F catheter. However, this can later be sized up (if required) via fluoroscopy when CC is mature and established. Peritoneum over the bladder is opened and bladder mobilized and anchored with stay sure to the abdominal wall. A detrusorotomy is then performed to create a detrusor defect in the posterior wall of the bladder and facilitate extravesical implantation of the appendix. An incision at the proximal end of the detrusorotomy is made and the tip of the appendix is opened to allow the Foley catheter to be inserted into the bladder lumen and the balloon is inflated. The length of detrusor defect should be a minimum of 4:1 ratio in comparison to the width of the appendix. The appendix is anastomosed to bladder mucosa with 50 PDS sutures followed by the detrusor wrap using interrupted 40 PDS sutures, making sure that some stitches do incorporate the appendix to maintain the tunnel length within the detrusor. The peritoneum is then reposited making sure the CC is extraperitoneal. VQ-plasty is performed to fashion a skin lined exit site for the CC. The CC is accessible for CIC typically after 6 weeks once it is mature.

### Post-operative Phase

**Figure 3:**
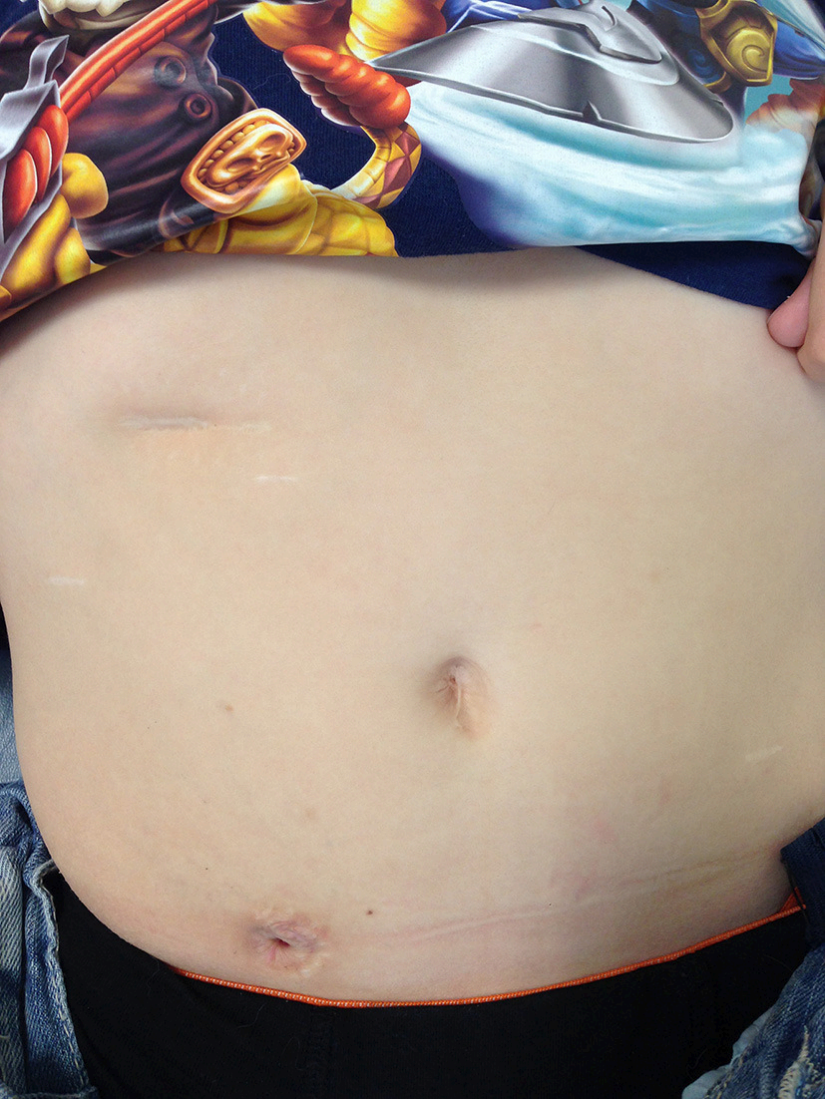
Postoperative appearance of a child 6 months after creation of CC.

All port sites are infiltrated with local anesthetic and none of the patients in this series have had epidural catheter for pain relief. The patients are allowed liquids the same afternoon with full feeds established shortly thereafter. The patients are discharged home with Foley catheter *in situ* via the CC. The Clinical nurse specialist supervises home care and starts CIC in ~6 weeks postop. A typical postoperative appearance of the CC approximately 6 months after the procedure is shown in Figure 3.

## Results

In this series, 18 children underwent procedure “Robotic approach to creation of CC.” The demographic details for each of these patients are as shown in Table 1. All procedures attempted by Robotic approach were successfully completed and there were no open conversions. Literature search produced 6 relevant and comparable published reports summarized in Table 2, all retrospective studies from a total of 6 centers across USA. There is one multicenter series reporting an experience of 88 cases involving 5 tertiary children's hospital (12). Three papers highlighted in gray within Table 2 are progressive reports over time from the same center in Chicago (led by Gundeti) (8–10). There is only one comparable series of CC procedure alone (7); all others have a mix of concomitant enterocystoplasty (EC). This makes the comparison challenging; nevertheless, the author has made an attempt to draw relevant conclusions from all the possible literature search yields.

**Table 1:** Summary of the author's experience with Robotic approach to creation of CC.

		**Comments**
No of cases (Gender)	18 (8M:7F)	Consecutive cases with Robotic approach
Median age at op (IQR)	10.75 (6.9–16.5)	Clinical indication–neurogenic (10) valve bladder (3) and others (5)
No of ports	3	Additional Step port at exit site to retrieve channel
Median OT (IQR)	197 min (131–295)	4 concomitant procedures−1 nephroureterctomy; 1 caecostomy button for bowel management; 1 proximal ureter reimplant with distal ureter as CC; 1 detrusorotomy (autoaugmentation)
Exit site VQ plasty	18	4 had caecostomy button *in situ* pre-op and 1 had concomitant caecostomy button inserted during this episode.
Channel size (Foley catheter)	10 F−1	10 F channel was later upscaled to 12 F by interventional radiology team after 4 months.
	12 F−13	
	14 F−4	
Channel conduit	14 appendix and 4 distal ureter	(When Ureters used for CC- 1 had nephroureterectomy and 3 others had proximal ureteric reimplant)
Conversion to open	0	
Median LOS (IQR)	2.75 (2–6)	Comparative open cases LOS 5.8 days
Complications post op	1	1 Wound infection at exit site; managed conservatively with antibiotics; No revision of CC in this series.
Median FU	27.3 m	Longest FU is 60 m; in total first 13 patients have a median FU of 36.6 months. Last 5 patients have relatively short FU of < 12 m.
Stomal stenosis	0	All patients use ACE stopper post op until channel matures in few months and CIC pattern established
Channel patency	18	3 patients had difficulty in accessing channel at entry into bladder; endoscopy confirmed no issues and 12 F Foley inserted easily. No sub-fascial channel issues.

*CC, Catheterisable channel; FU, Follow up; OT, Operating time; LOS, Length of stay in hospital post-op*.

**Table 2:** Summary of published articles on robotic approach to CC.

**References**	**CC**	**EC**	**No of CC cases(MedianAge at surgery)**	**Summary of findings (All retrospective reviews from USA and vast majority of cases in these series had umbilical exit site stoma)**
Nguyen et al. (7)	Yes	No	10 (11.9 y)	Mean OT 323 min; one conversion to open; Median Hosp stay 5 days; Median FU 14.2 m; one open revision due to urine leakage post op; minor channel incontinence 2 cases. Comparison with open cases-no difference
Wille et al. (8)	Yes	Yes (5)	11 (10.4 y)	Reports from same center over time; mean OT 494 min; 639 with EC and 347 min without EC; Median FU 24.2 m; Robot advantages: reduced hospital stay and eliminates epidural. Minimum recommended detrusor wrap length 4 cm. Appendix on anterior wall if without EC; otherwise posterior wall.
Famakinwa et al. (9)	Yes	Yes (10)	18 (11.7 y)	
Murthy et al. (10)	Yes	Yes (15)	11 (11 y)	
Grimsby et al. (11)	Yes	Yes	39 (9.1 y)	Comparison of complications between open (28) cases; 54% EC in open cases and 3% in Robotic; Median FU 2.7 y; no significant difference in complication rates between open and robotic; 3 Clavien III complications in Robotic series.
Gundeti et al. (12)	Yes	Yes (15%)	88 (10.4 y)	Multi center series; Mean FU 29.5 m; LOS 5.2 days; 29.5% complication rate; 6 Clavien III; 12% CC revision rate; Mean detrusor length 3.9 cm ± 1.0.

*CC, Catheterisable channel; EC, Enterocystoplasty; FU, Follow up; OT, Operating time; LOS, Length of stay in hospital post-op. Gray shaded rows indicate progressive over time reports from same institution*.

Salient features from the author's series are the following:

- Prospectively collected and analyzed data from a single surgeon series.
- Exclusive report of Robotic approach to creation of CC without EC.
- Median Operating time (OT) 197 min, much lower than 323 min from comparable series (Others have reported mean OT as 347 min for CC alone).
- All patients in the author's series have had their CC exit site stoma sited at the iliac fossa.
- No conversion to open and no immediate postoperative complications from the CC except one wound infection.
- Median LOS is 2.75 days; lower than reported multicenter series at 5.2 days. Comparatively, average LOS for open cases in the authors institution was 5.8 days. As per PLICS data, average bed occupancy in the children's ward is ~£300/day. Therefore, with an average saving of £1,000 per patient episode from this series, that makes a saving of ~£15–18 K for the hospital by using the Robotic approach in this series alone.
- Median FU is 27.3 months with the longest FU at 60 months and the average for the first 13 cases is 36.6 months i.e., over 3 years. All channels are patent currently with no incidence of exit site stomal stenosis. All patients use ACE stopper, the use of which has eliminated stomal stenosis as reported by the author in a previous publication many years ago (13).
- Experience with using ureter in selected cases for CC conduit; out of 4 children with ureteric mitrofanoff (all boys), 2 of them (50%) complain of transient pain when catheter enters the bladder.
- Three children have had some difficulty in accessing the CC at the very end toward its entry into the bladder at variable periods during FU. They underwent endoscopy to evaluate the channel ensuring patency and admitting 12 F catheter easily. One child who had 10 F catheter intraoperatively had interventional radiology support to upscale the catheter to 12 F, 4 months after the procedure.

## Discussion

This is a report of 18 consecutive cases with Robotic approach to creation of CC and is the first such report from a center outside the USA. Much of the published experience with the use of robot in pediatric urology is with pyeloplasty and upper tract procedures such as nephrectomy or heminephrectomy (3, 4, 14–17). Increasingly, the indications are expanding with reports on the use of robot for ureteric reimplantation (18–20).

The first description of the use of robot for creation of CC is a case report in 2004 (21). It then took a further 5 years before the publication of a series from a single center (7) and literature search for similar experience with the Robotic approach yields only a handful of published material, testifying that the experience with the use of robot for bladder reconstructive surgery is limited to few centers at this point in time. Moreover, the published material from some of the centers have a combination of EC together with CC, making the process of drawing definite conclusions at best challenging. Nevertheless, there are important points to highlight from the collective experience with common themes, variations in technique, and reported outcomes.

There is not a lot to dwell on indications for the procedure, fairly well-recognized and similar with the author's experience and published literature; and the age range at which surgery is performed is well-matched.

### Technical Aspects of the Creation of CC

In children, port placements are key to successful completion of the procedure by the robotic approach, because the recommended 8–10 cm distance between ports in adults is not realistically achievable. The author routinely places three ports, one for the camera, and two working ports corresponding to three arms of the robot as is the preference for other surgeons in published literature except for Grimsby et al. (11) where they prefer a 4th arm is deployed for assistance. In a pediatric program, the 4th arm is pretty much redundant for majority of the procedures. Some suggest use of a 5 mm laparoscopic port to exchange sutures (7), but the author relies on the assistants to exchange sutures via the working port, which of course needs excellent team effort. The author cannot emphasize enough the role of a well-trained first assistant as regards efficiency and minimizing the need for additional port in an already cramped set up. The benefits of side docking in pediatric robotic program and alluded to in the technical aspects earlier in the report has been published elsewhere (6).

One important difference in this series is where the ports are placed. The author places the camera 12 mm port at the midline in the epigastric region and two ports on the hypochondrium to stay further away from the target organs, bladder, and appendix. In comparison, all the other surgeons (in published literature) place the ports much closer to the target organs with the camera port at the umbilicus. It is also worth noting that the ureter has been used as a conduit in selected cases in this series, the use of which has raised some observations and is further explained in the outcomes section Follow up and Outcomes.

It appears in those published series in Table 2; the exit site is preferentially the umbilicus whilst the author routinely sites the exit stoma in the iliac fossa. The use of step port to retrieve the conduit onto the exit site is another variation in the author's practice. It is difficult to know for sure, but it appears that the author's technique described earlier has contributed to a much lower median OT at <200 min compared to >300 min in other reported series (7, 22).

Incidence of concomitant EC is high in the reports especially from Chicago group (9, 10, 22) and the multicenter report (12) where there is a 15% incidence of EC and 39% incidence of bladder neck procedures (BNP). Therefore, this series is matched and comparable only to the report from Boston group Nguyen et al. (7) with regards to creation of CC without concomitant EC. It is interesting to note when comparing open and Robotic approach to CC, Grimsby et al. in their series report an incidence of concomitant EC is 54% in the open group vs. 3% in the Robotic group (11). This could be explained by the complexity of adding EC to the procedure at the same setting, given that the Chicago group led by Gundeti report mean OT at 623 min with EC compared to 347 with CC alone (8–10). To put into perspective, that is over 10 h of operating with EC, one of the main reasons for the limited experience with such complex reconstructive procedures.

In this series, the site of implantation of the CC in to the bladder is in the posterior wall with the bladder hitched up ensuring straight run off to the exit site. Interestingly, Famakinwa et al. from the Chicago group (9) mention the difference in implantation site if the CC is performed concomitantly with EC; the anterior wall is preferred if CC is done alone and the posterior wall if combined with EC.

### Postoperative Phase and Complications

Murthy et al. have highlighted the advantages of using the robot in this procedure with reduced hospital stay and eliminating the need for epidural (10), which the author is in complete agreement.

The average LOS is much lower in the author's series at 2.75 days compared to 5.2 days in the multicenter series (12). In the healthcare setting within the UK, LOS is crucial to costs and by reducing the LOS with robotic approach compared to open (2.7 vs. 5.8 days as explained in the results section), significant savings have been made in the author's series. This is important when it comes to working collaboratively with the management to realize the feasibility of a robotic program as also observed by others (23). Differences in healthcare worldwide could explain the paucity of reports linked to affordability from centers outside USA.

In this series, there has been no conversion to open and no major immediate postoperative complications except for an exit site wound infection in one case unlike a reported 29.5% complication rate with 6 Clavien III episodes from the multicenter series (12). It must be noted that they had concomitant 17% EC and 39% BNP. However, Grimsby et al. also reported 3 Clavien III complications in the Robotic group of their series (11). Nguyen et al. report one conversion to open in their series with 10 cases with one post-op urinary leak (7).

### Follow Up and Outcomes

The mean FU is 27.3 m in this series and is comparable to the FU in the multicenter study of 29.5 m (12). The longest FU in this series is 60 months and the first 13 cases have over 3 years FU. This should be sufficient time to look at outcomes given that majority of the revisions occur in the first 24 months (24, 25). Although this point is highlighted by Nguyen et al. in their report, their FU of just 14.2 m is relatively short (7). There have been no revisions of the CC or the exit site in this series. This is significant compared to 12% CC revision rate in the multicenter study (12). Grimsby et al. report ~30% reoperation rates in both open and Robotic groups, although the reasons behind them is varied (11). They also have had 10% incontinence rates in the robotic group. It is difficult to know from the available reports whether the CC incontinence was technique related or due to other factors such as bladder dynamics.

There was no incidence of incontinence attributable to the technical aspects of CC in this series and it is worth mentioning the emphasis by the author on a minimum ratio of 4:1 when constructing then channel. The length of detrusor wrap is recognized as an important factor by others with a recommended length of 4 cm to ensure continence (8, 12). The author prefers to relate it to the width of the appendix akin to the principle with a ureteric reimplantation procedure.

It is important to mention that 4 boys in this series have had distal ureter as a CC conduit and 2 of them (50%) have reported considerable transient pain at point of entry into the bladder and 2 other boys (50%) in this series use the ureteric CC with no discomfort. This is also the author's experience with open cases and therefore now re-evaluating the use of ureter.

The author has published previously his experience with ACE stopper in the postoperative period (13) and this practice has continued over the years with stomal stenosis virtually eliminated and as is the case in this series. Wille et al. have reported 3 exit site revisions in their series of 11 cases (27%) (8). There is no incidence of CC access issues at sub-fascial plane in this series or other series with Robotic approach compared to reports by Indiana group who have the largest experience with CC using the open approach (26). Plausible explanation could be the direct alignment of the channel from within the abdomen with the robotic approach.

### Summary

This is the first report of Robotic approach to CC from outside of USA with comparable outcomes and together with the collective experience from published literature albeit from few centers establishes the role of Robotics in creation of CC. There are common themes with some variation in technical aspects but the main advantages being reduced hospital stay and avoiding the need for invasive pain relief such as epidural compared to open approach. The series in this report has the lowest mean OT and LOS, both contributing to cost savings from a healthcare economic model point of view. The experience with robotic approach is limited to tertiary centers currently but there is sufficient expertise to allow other aspiring centers to take advantage of and avail the proctorship to facilitate safe introduction of complex reconstructive surgery into their individual Robotic program (27). This can only enhance the quality of care provided over time within pediatric urology.

## Ethics Statement

This study material is part of an audit within the department. Informed consent for figures was obtained as per institution guidelines.

## Author Contributions

The author confirms being the sole contributor of this work and has approved it for publication.

### Conflict of Interest Statement

The author declares that the research was conducted in the absence of any commercial or financial relationships that could be construed as a potential conflict of interest.
